# Supplementary material for: Investigation of Bar-seq as a method to study population dynamics of Saccharomyces cerevisiae deletion library during bioreactor cultivation
Source: Microb Cell Fact. 2020 Aug 18;19:167. doi: 10.1186/s12934-020-01423-z (PMC7437010; doi:10.1186/s12934-020-01423-z)
Supplement: Supplementary file 1 — Additional file 1. Supplementary figures and tables. [file 12934_2020_1423_MOESM1_ESM.docx]

Additional Information

Investigation of Bar-seq as a method to study population dynamics of Saccharomyces cerevisiae deletion library during bioreactor cultivation

Maren Wehrs^1,2^*, Mitchell G. Thompson^1,2^*, Deepanwita Banerjee^1,2*^, Jan-Philip Prahl^1,3^, Norma M. Morella^,4^, Carolina A. Barcelos^1,3^, Jadie Moon^1,2^, Zak Costello^1,2,5^, Jay D. Keasling^1,2, 7,8,9,10^, Patrick M. Shih^2,11,12^, Deepti Tanjore^1,3,  +^, Aindrila Mukhopadhyay^1,2,11, +^

*Denotes equal contribution

^+^ Corresponding author: dtanjoore@lbl.gov and amukhopadhyay@lbl.gov

^1^Biological Systems and Engineering Division, Lawrence Berkeley National Laboratory, Berkeley, CA 94720, ^2^Joint BioEnergy Institute, Lawrence Berkeley National Laboratory, Emeryville, CA 94608, ^3^Advanced Biofuels and Bioproducts Process Development Unit, Lawrence Berkeley National Laboratory, Emeryville, CA 94608, ^4^,  Fred Hutchinson Cancer Research Center, Seattle, WA 98109, USA ^5^Department of Energy Agile BioFoundry, Emeryville, CA 94608, ^6^Department of Plant and Microbial Biology, University of California, Berkeley, CA 94720, USA, ^7^Department of Bioengineering, University of California, Berkeley, CA 94720, USA, ^8^Department of Chemical and Biomolecular Engineering, University of California, Berkeley, CA 94720, USA, ^9^The Novo Nordisk Foundation Center for Biosustainability, Technical University of Denmark, Denmark, ^10^Synthetic Biochemistry Center, Institute for Synthetic Biology, Shenzhen Institutes for Advanced Technologies, Shenzhen, China ^11^Environmental Genomics and Systems Biology Division, Lawrence Berkeley National Laboratory, Berkeley, CA 94720, ^12^Department of Plant Biology, University of California-Davis, Davis, CA 95616, USA


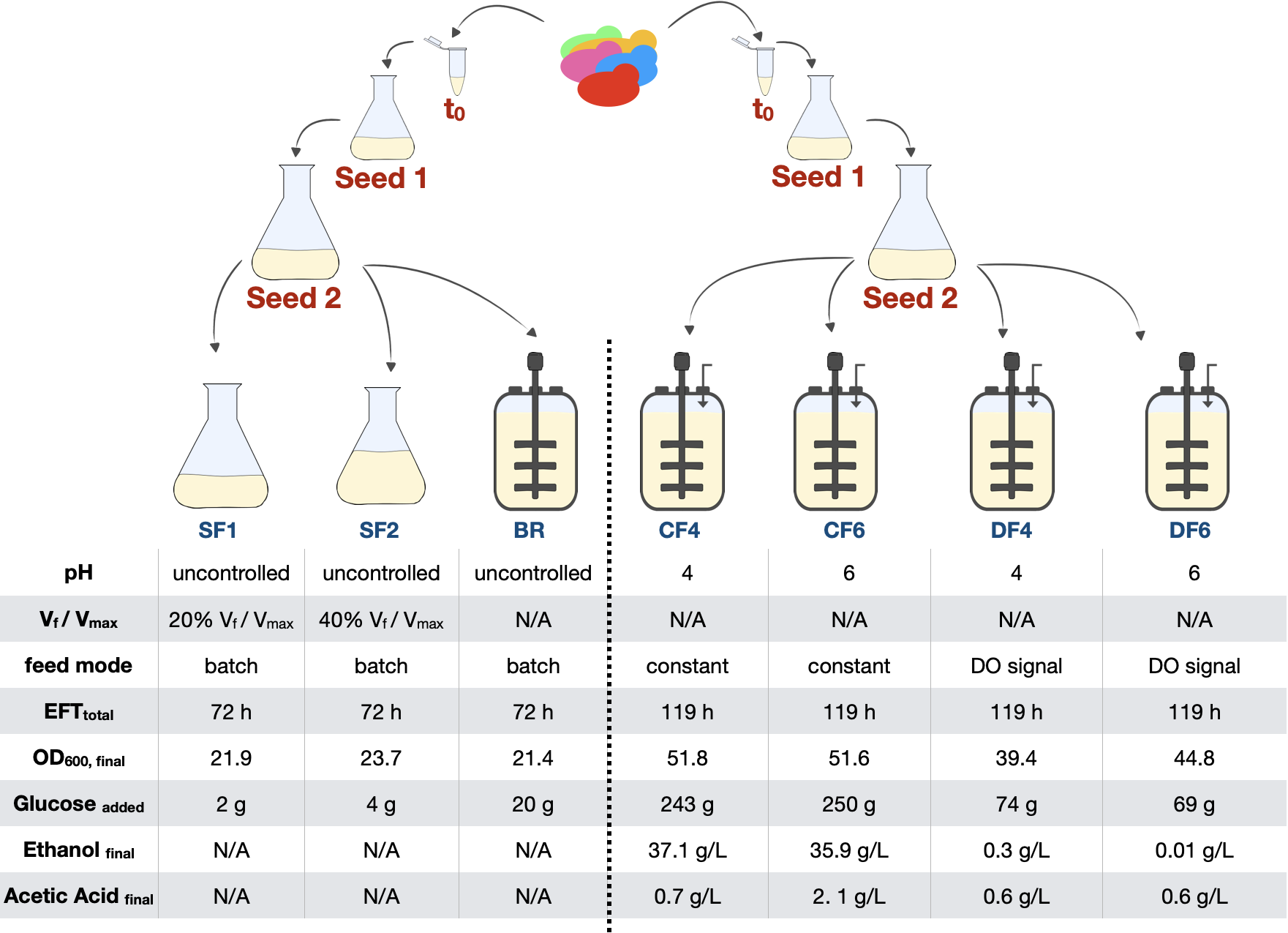


**Figure S1:** **Overview of all conditions tested in this study.** Growth competition experiments were done in two sets, each consisting of two seed train stages (Seed 1 and Seed 2) followed by different main cultivation environments. Set 1 (left) varied in vessel architecture that included batch bioreactor (BR) and shake flasks with different culture volumes (SF1 and SF2). Set 2 (right) varied in cultivation parameter setting that included four fed-batch mode bioreactors (CF4, CF6, DF4 and DF6) with two different feeding modes and two different pH.

| A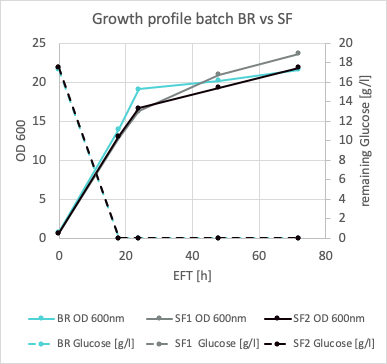  B 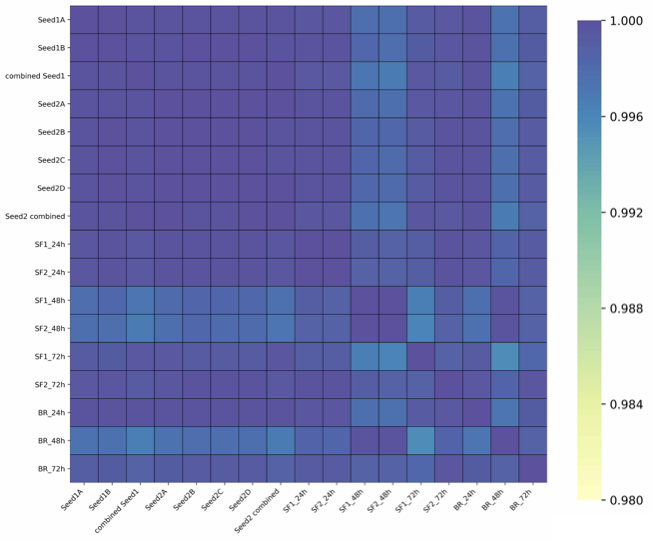 | C   \| 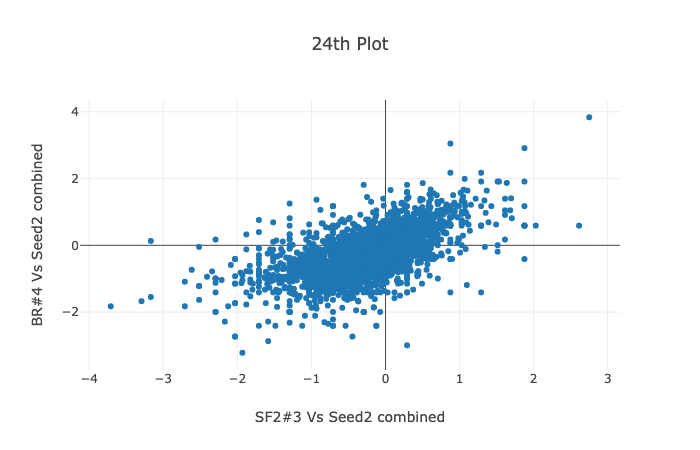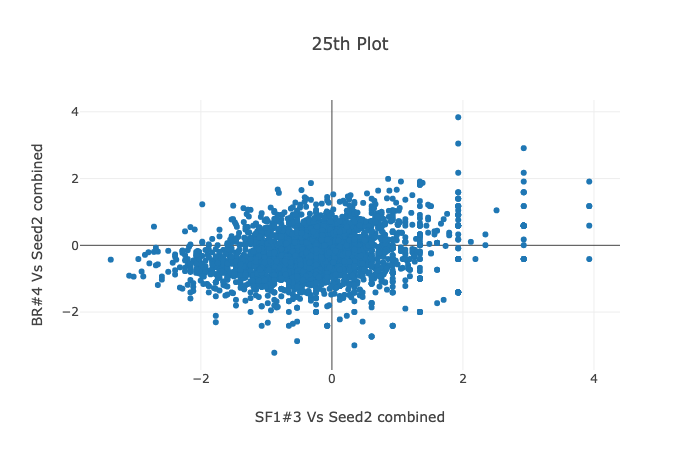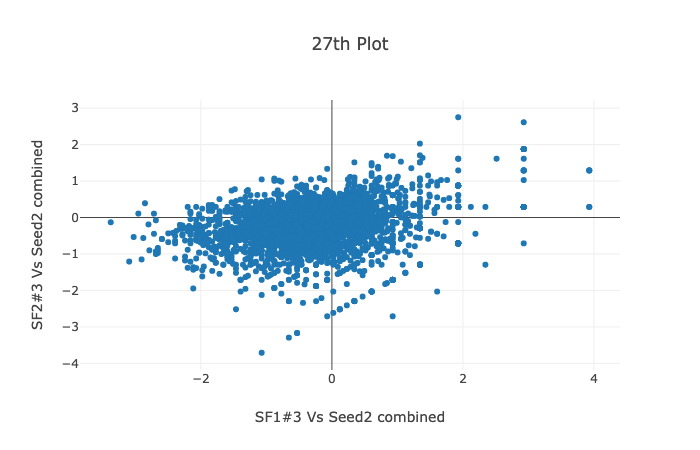 \| \| --- \| |
| --- | --- | --- |

**Figure S2: Similar population dynamics in Set 1 between shake flask and batch bioreactors.** Growth (represented by OD_600_) and glucose consumption (in g/L) profiles (A) for batch bioreactor (BR) and shake flasks (SF1 and SF2) during the entire cultivation time period. B)Heatmap of the Pearson correlation matrix (B) and scatter plots (C) of mutant counts for all three cultivation experiments in Set 1 growth competition experiments in comparison to seed trains.

| 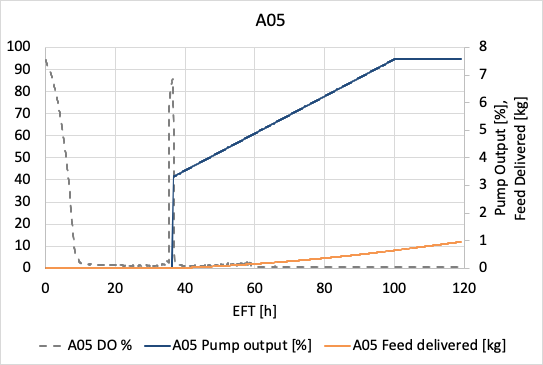 | 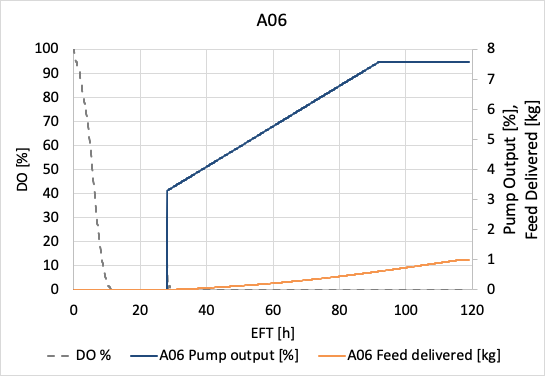 |
| --- | --- |
| 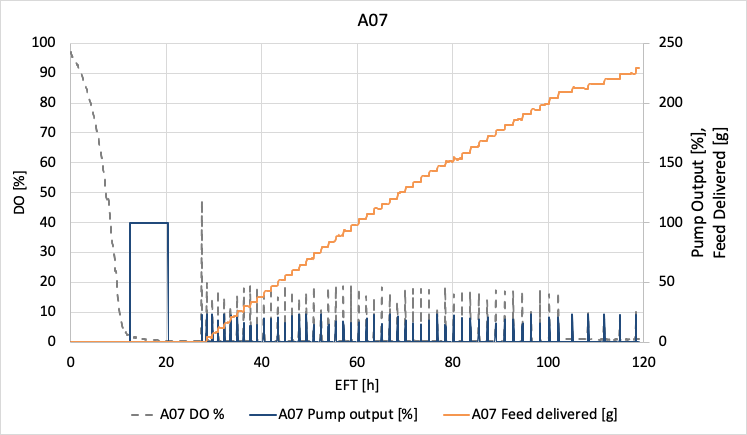 | 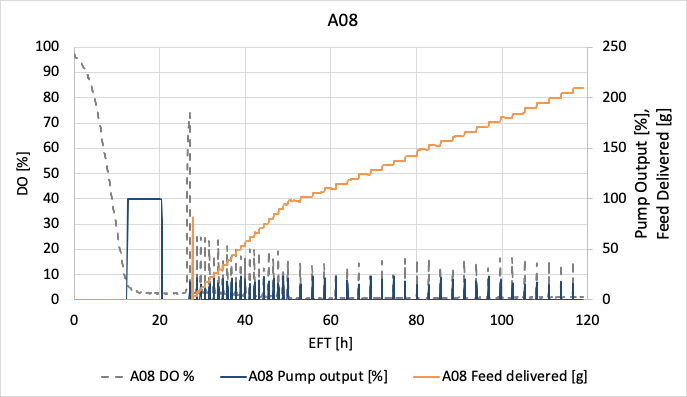 |

**Figure S3**: **Feeding profiles and pump output of each fed-batch bioreactor over time.** In all conditions, the feed was started upon an initial spike in dissolved oxygen (DO, grey line), indicating the consumption of available carbon sources from the batch media. For CF4 and CF6, a linear feed was implemented which resulted in linear addition of glucose (orange line) to the cultivation broth. For DF4 and DF6, the feeding pump activity (dark blue) was programmed to respond to changes in the DO (DO stat) and thus reacted to the metabolic requirements of the culture. Please note that the pump output 100 % between 18-20 h is an artefact and no feed solution was supplied to the tank during this feed controller test.


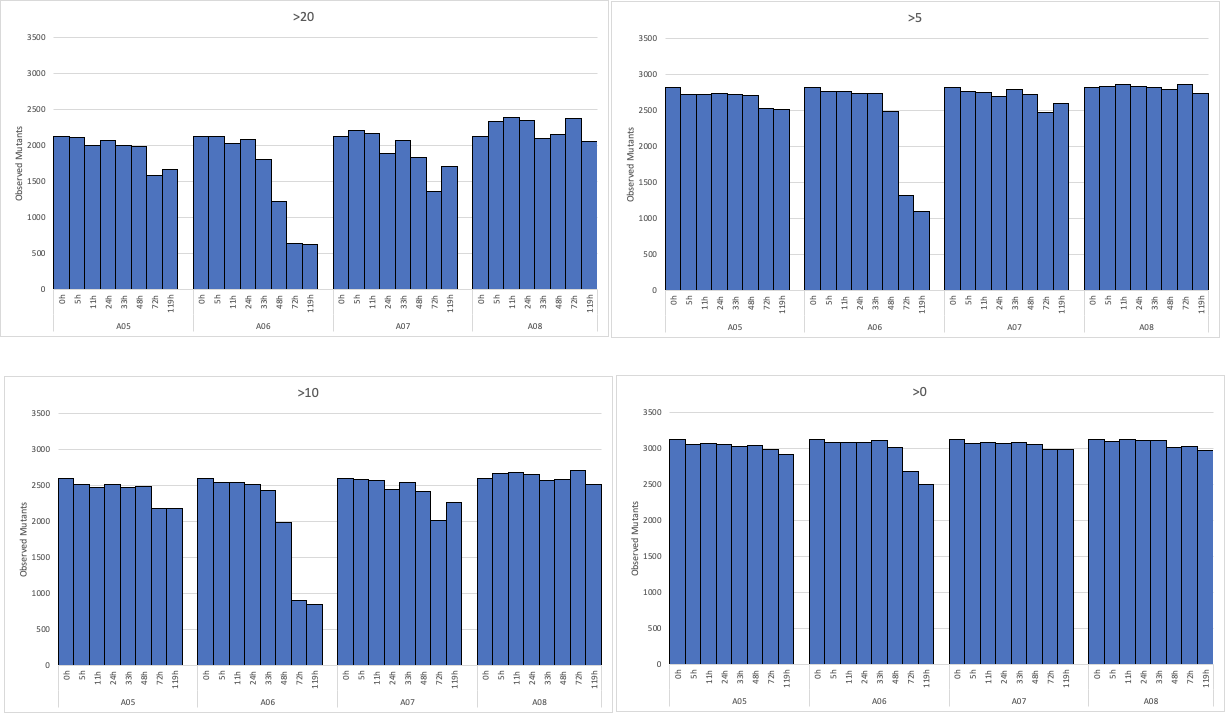


**Figure S4:** **Trends observed for mutants in each fed-batch bioreactor in Set 2 are independent of the mutant count threshold.** Population diversity of each fed-batch bioreactor over time using different cut off values (>20, >10, >5 and >0) in Seed 2 for the analysis. A06 had a completely different population dynamics compared to the other three bioreactors.

**A B**


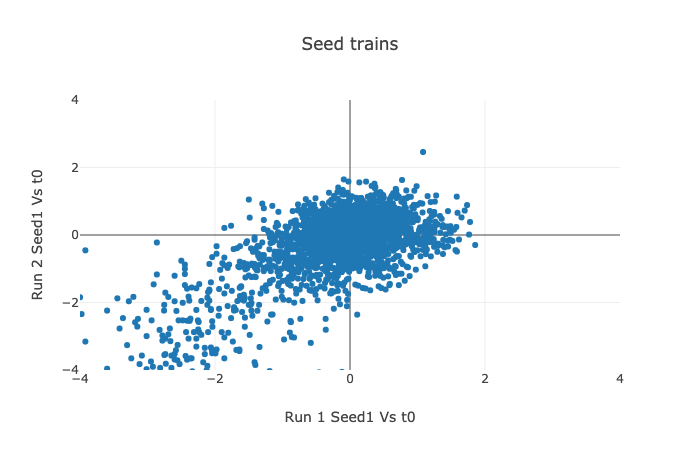

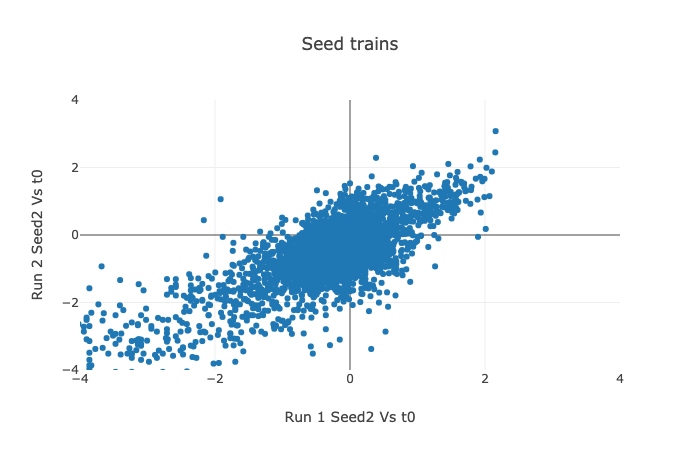


**C**

**Figure S5**: **Similar population dynamics between t0 and different seed trains.** Scatter plots (A and B) and heatmap of the Pearson correlation matrix (C) of mutant counts for t0 and individual seed trains across Set 1 and Set 2 growth competition experiments.
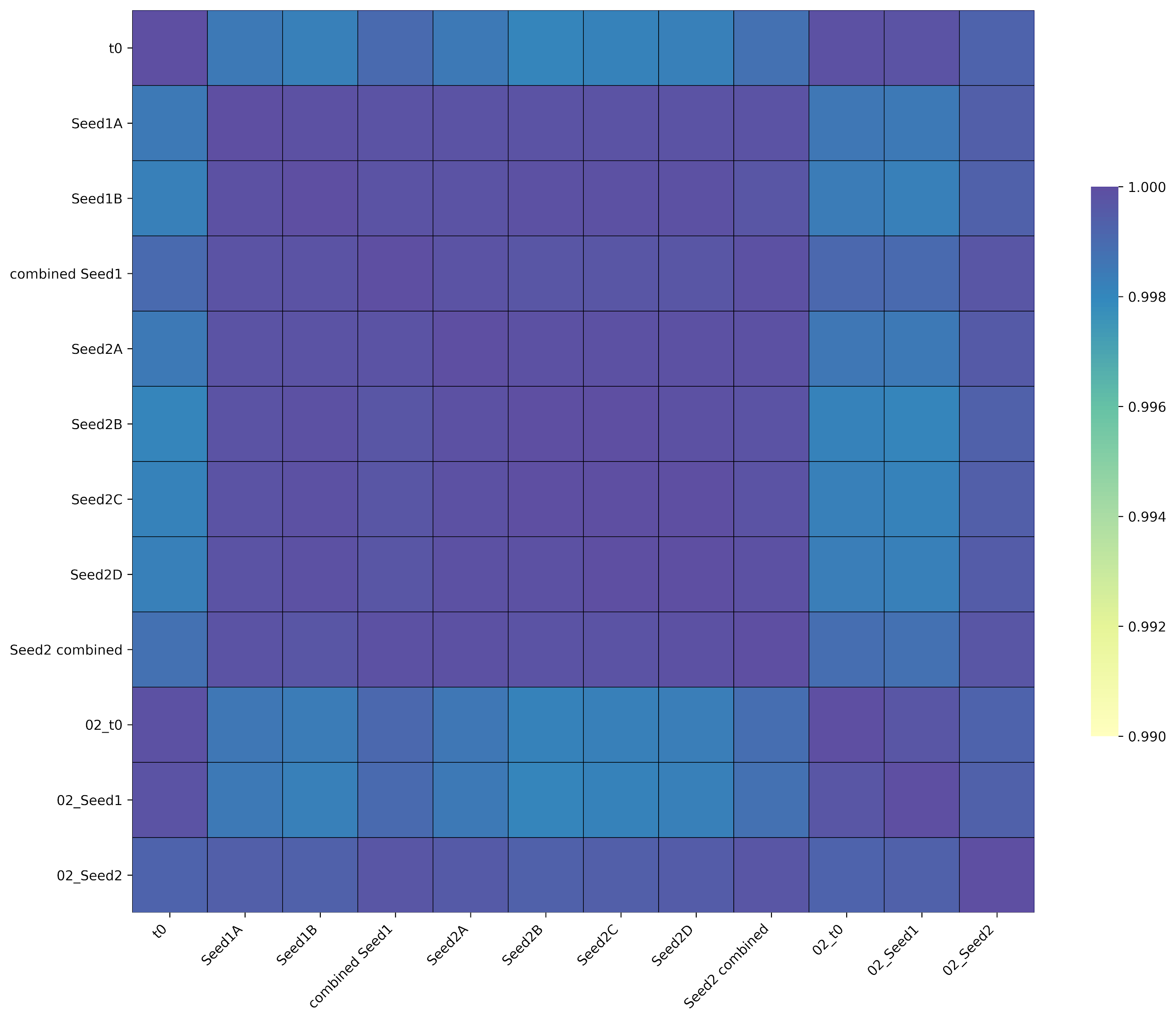


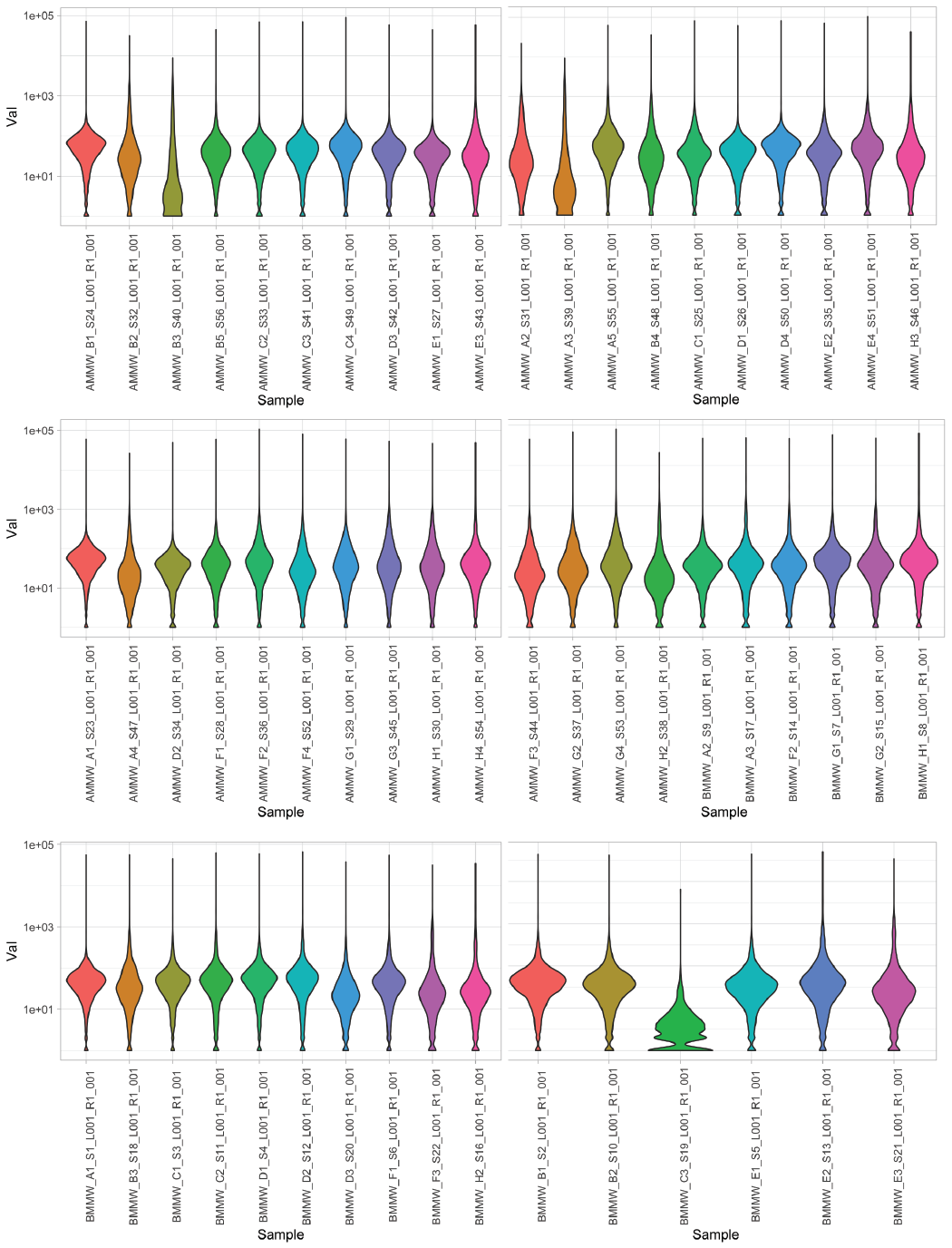


**Figure S6:** **Distribution of mutant barcode counts across all samples.** Violin plots show the distribution of counts per genes across all experiments.

**Table S1: Number of strains for which fitness was measured in Set 2 and had at least 10 counts**

| **-48** | **-24** | **0** | **Time (h)** | **5** | **11** | **24** | **33** | **48** | **72** | **119** |
| --- | --- | --- | --- | --- | --- | --- | --- | --- | --- | --- |
| **t0** | **Seed 1** | **Seed 2** | **CF4** | 2425 | 2415 | 2431 | 2409 | 2391 | 2132 | 2151 |
| 2562 | 2562* | 2562 | **CF6** | 2440 | 2443 | 2440 | 2378 | 1976 | 896 | 844 |
|  |  |  | **DF4** | 2467 | 2460 | 2392 | 2430 | 2358 | 2028 | 2204 |
|  |  |  | **DF6** | 2492 | 2502 | 2513 | 2445 | 2405 | 2465 | 2372 |

*Seed 1 had 2542 mutant barcode strains with at least 10 counts.

**Table S2: Summary of important genetic targets with the annotated gene functions**

| **Gene Name** | **Systematic Name** | **Gene Function** | **GO Terms** | **GO Term Description** | **Selection** |
| --- | --- | --- | --- | --- | --- |
| ***mck1*** | YNL307C | Meiosis and Centromere regulatory Kinase | GO:0000070  GO:0006303  GO:0006468  GO:0006974  GO:0007165  GO:0016310  GO:0018105  GO:0030437  GO:0034605  GO:0043086  GO:0044257  GO:0051321  GO:0071472 | Stress Response | Enriched For |
| ***mrk1*** | YDL079C | Mds1p Related Kinase | GO:0006468  GO:0007165  GO:0016310  GO:0034605  GO:0042176  GO:0071472 | Stress Response | Enriched For |
| ***msh4*** | YFL003C | MutS Homolog | GO:0000712  GO:0007131  GO:0051321  GO:0001302 | Stress Response | Enriched For |
| ***msn4*** | YKL062W | Multicopy suppressor of SNF1 mutation | GO:0001306  GO:0006338  GO:0006357  GO:0042594  GO:0043619  GO:0045944 | Oxidative Stress | Enriched For |
| ***rim11*** | YMR139W | Regulator of IME2 | GO:0006468  GO:0006508  GO:0007165  GO:0016310  GO:0030437  GO:0034605  GO:0071472 | Stress Response | Enriched for |
| ***ygk3*** | YOL128C | Yeast homolog of Glycogen synthase Kinase | GO:0006468  GO:0006508  GO:0016310  GO:0034605  GO:0051726  GO:0071472 | Stress Response | Enriched For |
| ***aim25*** | YJR100C | Altered Inheritance of Mitochondria | GO:0017121  GO:0034599  GO:0034605  GO:1903147 | Lipid Transport | Selected Against |
| ***atg2*** | YNL242W | AuTophaGy related | GO:0000045  GO:0000422  GO:0006914  GO:0015031  GO:0030242  GO:0032258  GO:0034727  GO:0044804  GO:0044805  GO:0061709 | \| Autophagy \| \| --- \| | Selected Against |
| ***atg3*** | YNR007C | AuTophaGy related | GO:0000045  GO:0000422  GO:0006501  GO:0006914  GO:0015031  GO:0032258  GO:0034727  GO:0044804  GO:0044805 |  | Selected Against |
| ***atg31*** | YDR022C | AuTophaGy related | GO:0000422  GO:0000741  GO:0006914  GO:0007059  GO:0015031  GO:0034727  GO:0044805 |  | Selected Against |
| ***atg4*** | YNL223W | AuTophaGy related | GO:0000422  GO:0006501  GO:0006508  GO:0006914  GO:0015031  GO:0032258  GO:0034727  GO:0044805  GO:0051697 |  | Selected Against |
| ***atg8*** | YBL078C | AuTophaGy related | GO:0000422  GO:0006888  GO:0006914  GO:0006995  GO:0015031  GO:0016236  GO:0016241  GO:0032258  GO:0034497  GO:0034629  GO:0034727  GO:0044805  GO:0061025  GO:0061709  GO:0071211  GO:1905153 |  | Selected Against |
| ***atg9*** | YDL149W | AuTophaGy related | GO:0000045  GO:0000422  GO:0006914  GO:0015031  GO:0032258  GO:0034497  GO:0034727  GO:0044805  GO:0061709 |  | Selected Against |
| ***boi2*** | YER114C | Bem1 (One) Interacting protein | GO:0000920  GO:0001881  GO:0007015  GO:0007032  GO:0007118  GO:0042147 | Endosomal Transport | Selected Against |
| ***cit3*** | YPR001W | CITrate synthase | GO:0005975  GO:0006099  GO:0019629 | TCA | Selected Against |
| ***cmr1*** | YDL156W | Changed Mutation Rate | GO:0006974  GO:2000001 | Stress Response | Selected Against |
| ***cne1*** | YAL058W | CalNExin and calreticulin homolog | GO:0006457  GO:0030433  GO:0030968 |  | Selected Against |
| ***cox8*** | YLR395C | Cytochrome c OXidase | GO:0006119  GO:0006123  GO:0055114 | TCA | Selected Against |
| ***cta1*** | YDR256C | CaTalase A | GO:0000302  GO:0001315  GO:0006979  GO:0042542  GO:0042744  GO:0055114  GO:0098869 | Oxidative Stress | Selected Against |
| ***dic1*** | YLR348C | DIcarboxylate Carrier | GO:0006817  GO:0006835  GO:0008272  GO:0015709  GO:0015729  GO:0035435  GO:0071422  GO:0071423  GO:1902356  GO:1902358 |  | Selected Against |
| ***did2*** | YKR035W-A | Doa4-Independent Degradation | GO:0006623  GO:0007034  GO:0015031  GO:0032509  GO:0032511  GO:0045324  GO:1904902 | Endosomal Transport | Selected Against |
| ***dpb4*** | YDR121W | DNA Polymerase B (II) subunit | GO:0001302  GO:0006260  GO:0006261  GO:0006272  GO:0006974  GO:0031507  GO:0042276  GO:0042766  GO:0043044  GO:0070868  GO:0090305 | Stress Response | Selected Against |
| ***drs2*** | YAL026C | Deficiency of Ribosomal Subunits | GO:0000749  GO:0006886  GO:0006892  GO:0006897  GO:0015914  GO:0032456  GO:0045332  GO:0140331 | Lipid Transport | Selected Against |
| ***esa1*** | YOR244W | Subunit of the Piccolo NuA4 histone acetyltransferase complex | GO:0000183  GO:0001207  GO:0006281  GO:0006325  GO:0006354  GO:0006355  GO:0006357  GO:0016239  GO:0016573  GO:0018394  GO:0032968  GO:0043967  GO:0045892  GO:0045944  GO:0051726 | Stress Response | Selected Against |
| ***flr1*** | YBR008C | FLuconazole Resistance | GO:0006855  GO:0015903  GO:0042908  GO:0055085  GO:1990961 | Oxidative Stress | Selected Against |
| ***gem1*** | YAL048C | GTPase EF-hand protein of Mitochondria | GO:0000001  GO:0007005  GO:0010821  GO:0015886  GO:0055091  GO:1990456 | Stress Response | Selected Against |
| ***gpd1*** | YDL022W | Glycerol-3-Phosphate Dehydrogenase | GO:0005975  GO:0006072  GO:0006116  GO:0006973  GO:0016558  GO:0046168  GO:0055114 | TCA | Selected Against |
| ***gph1*** | YPR160W | Glycogen PHosphorylase | GO:0005975  GO:0005977  GO:0005980 | TCA | Selected Against |
| ***idh1*** | YNL037C | Isocitrate DeHydrogenase | GO:0006099  GO:0006102  GO:0006537  GO:0008152  GO:0055114 | TCA | Selected Against |
| ***msc6*** | YOR354C | Meiotic Sister-Chromatid recombination | GO:0006310  GO:0007131  GO:0051321 | Stress Response | Selected Against |
| ***mxr1*** | YER042W | peptide Methionine sulfoXide Reductase | GO:0034599  GO:0055114 | Oxidative Stress | Selected Against |
| ***osh2*** | YDL019C | OxySterol binding protein Homolog | GO:0006869  GO:0006887  GO:0006897  GO:0015918  GO:0030011  GO:0034727  GO:0035621  GO:0120009 | Autophagy | Selected Against |
| ***pmt7*** | YDR307W | Predicted integral membrane protein whose biological role is unknown | GO:0006486  GO:0006493  GO:0008150  GO:0035269  GO:0071712 | Oxidative Stress | Selected Against |
| ***rad57*** | YDR004W | RADiation sensitive | GO:0000707  GO:0000722  GO:0000730  GO:0006281  GO:0006312  GO:0006974  GO:0010212  GO:0030491  GO:0042148  GO:0051321 | Stress Response | Selected Against |
| ***rfs1*** | YBR052C | Rad55 (Fifty-five) Suppressor | GO:0008150  GO:0055114 | TCA | Selected Against |
| ***ric1*** | YLR039C | RIbosome Control | GO:0006886  GO:0042147 | Endosonal Transport | Selected Against |
|  |  |  |  |  |  |
| ***rpl8b*** | YLL045C | Ribosomal Protein of the Large subunit | GO:0000470  GO:0002181  GO:0042254 | Ribosomal and Translational activity | Selected Against |
| ***rps0a*** | YGR214W | Ribosomal Protein of the Small subunit | GO:0000028  GO:0000447  GO:0000461  GO:0002181  GO:0006364  GO:0006407  GO:0006412  GO:0042254 | Ribosomal and Translational activity | Selected Against |
| ***sol3*** | YHR163W | Suppressor Of Los1-1 | GO:0005975  GO:0006098  GO:0009051 | TCA | Selected Against |
| ***ssm4*** | YIL030C | Suppressor of mrna Stability Mutant | GO:0001300  GO:0016567  GO:0030433 | Stress Response | Selected Against |
| ***stb3*** | YDR169C | Sin Three Binding protein | GO:0000432 | Oxidative stress | Selected Against |
| ***tim18*** | YOR297C | Translocase of the Inner Mitochondrial membrane | GO:0006915  GO:0006970  GO:0015031  GO:0034599  GO:0045039  GO:0046685  GO:0071806 | TCA | Selected Against |
| ***tsa1*** | YML028W | Thiol-Specific Antioxidant | GO:0000077  GO:0001302  GO:0006111  GO:0006457  GO:0006979  GO:0033194  GO:0034599  GO:0042262  GO:0043549  GO:0045454  GO:0055114  GO:0061077  GO:0071470  GO:0090344  GO:0098869 | Stress Response | Selected Against |
| ***ups3*** | YDR185C | UnProceSsed | GO:0015914  GO:0070584  GO:0120009 | Lipid Transport | Selected Against |
| ***vps30*** | YPL120W | Vacuolar Protein Sorting | GO:0000045  GO:0006661  GO:0006914  GO:0006995  GO:0015031  GO:0016236  GO:0030242  GO:0032258  GO:0034727  GO:0042147  GO:0045324 | Autophagy and Endosomal Transport | Selected Against |
| ***vps35*** | YJL154C | Vacuolar Protein Sorting | GO:0006886  GO:0015031  GO:0042147  GO:0045053 | Endosomal Transport | Selected Against |
| ***vta1*** | YLR181C | VpsTwenty Associated | GO:0006869  GO:0015031  GO:0032511  GO:0032781  GO:0045324 | Endosomal Transport | Selected Against |
| ***ybp1*** | YBR216C | Yap1-Binding Protein | GO:0034599 | Oxidative Stress | Selected Against |
| **-** | YLR255C | - | GO:0008150 | Oxidative Stress | Selected Against |
